# Dentists as Buyers

**Published:** 1910-01-15

**Authors:** George Wood Clapp


### DENTISTS AS BUYERS.

BY GEORGE WOOD CLAPP, D.D.S.

If dentists were as good buyers as the most of them are spenders, all would be wealthy. Most of them spend with free hands and happy hearts. But few seem to perceive that behind the glad spending must run a line of good business practice or the spending will sometime suddenly and permanently stop because there is nothing left to spend.

It is usually recognized among merchants that financial success comes very largely from intelligent buying of the supplies or merchandise necessary to the business, both as to the quality of goods bought *and the terms*. The shrewd merchandise buyer fights for an extra 2 per cent. or 1 per cent., or even ½ per cent., discount for cash. Offers at which the dentist would turn away in scorn are eagerly sought by him as the corner-stone of money making.

As this article is being written there returns from Europe a member of a New York firm which has made a very rapid success in its line. This member journeyed to Paris to get an extra discount, *not equal in per cent. to that quoted regularly to dentists*. He came back delighted because he had secured an additional 5 per cent, on conditions which will compel him to borrow many thousands of dollars to meet the payments. While this member was in Paris, the acting manager borrowed, at one time, $15,000 with which to discount the current bills, which he had no funds to pay.

It is much better business to borrow money with which to take all discounts at their maximum than to allow bills to go to maturity simply because one has not the money in hand to pay them when presented. The firm mentioned above has large and expensive show-rooms on Fifth Avenue in New York City. It is expected that the profits from discounts this year will pay the rent of these rooms.

Numberless incidents of this sort might be reproduced as the ordinary practice of the business world. Shrewd buying and prompt payments lay at the root of every mercantile success. Good buyers watch the pennies and the dollars watch themselves.

In some cases this intelligent form of buying has been adopted among dentists. But extensive investigation shows that it is only in rare cases. Most dentists are very poor buyers because they are very poor payers. They contract bills readily, but rarely provide ready funds for prompt payment. The discounts quoted them for prompt payment would be regarded by any shrewd merchant as very large. The merchant would immediately borrow of his bank the money to pay the bill and secure such discount; and of two merchants dealing side by side, the one who took such discounts could put the one who did not, out of business. But because the amount involved is not large, the dentist thinks it is not worth while to struggle for the funds and lets the bill run to maturity and perhaps longer.

Let us take some actual figures, that we may get at definite values. Reports from various sections of the country indicate that the average monthly purchases by dentists, exclusive of precious metals, amount to about $20. These figures were arrived at as follows: the managers of a number of depots each averaged twenty-five accounts, purposely excluding large accounts, and sent their averages to the writer of this article. The averages sent in from different sections of the country were then averaged and the final sum was as above. This figure then may be taken as representative.

Is it worth while for a dentist buying $20 per month to discount his bills? Isn't it more trouble than it is worth? Let us see.

Suppose the dentist puts that $20 out at interest. His annual return from any safe investment will not exceed 6 per cent., or $1.20. But let him use it to discount his monthly dental bill and he will get 40 cents discount the first month. During the month he will sell to patients the materials he purchased with the $20, and should so conduct his collections that he will have the $20 again in hand plus his profits when the materials are used. He discounts that month's bill and gets 40 cents more. At the end of the year that $20 has earned $4.80, or 24 per cent. on the capital. This is four times the amount of interest received from any safe investment and six times the amount a bank pays.

Some dentists figure that they have more than $20 invested because they pay twelve bills, but the accepted form of figuring among bankers and other financiers is that $20 is the total investment, because one $20 bill can be made to carry the business. That is, $20 pays for the month's purchases. The sale of these purchases during the month produces again the $20 necessary to pay for a new supply and the sale of his services produces the dentist's profits.

The trouble is that dentists too often scorn so humble a sum as this $4.80 gotten in installments of 40 cents each. It is just this scorn of small amounts and hatred of the trouble necessary to get them that renders their business conduct so unprofitable. Forty cents pays only for small things, an extra plugger or something like that. But a practice buying only this much is a small business, and small leaks may readily drain away the entire capital.

Furthermore, paying promptly requires that the dentist collect his bills promptly.

And in the failure to do this lays the secret of the whole trouble. Our professional predecessors were usually poor collectors, and we are their true descendants. From lack of business sense or of the nerve to put it into practice, we are mostly wretched collectors. We labor faithfully, we use up the supplies we purchase, but when it comes to claiming prompt payment as our right we lose our nerve and too often let the patient pay when he will. Of course it is then hard for us to pay promptly. And thus we are ground between the upper millstone of those who owe us and the lower millstone of those whom we owe. A superficial examination of our assets shows that most of us are ground exceedingly fine. And the fault is ours—ours—ours.

The discount quoted to the dentist for prompt payment at the end of the month is the smallest of several quoted to him. The others, when figured out, make possible such savings as are the wonder of all merchants. And they look with little less than scorn on the man who neglects them. They think him a very poor business man indeed; in their eyes he deserves to fail.

To the dentist who buys $25 worth of supplies (other than precious metals) at one time, for cash, Dental Depots quote 5 per cent. discount. The dentist we are considering buys $240 worth of supplies of such a year. If an extra discount can be obtained by buying this amount in purchases of $25 instead of $20, business sense would indicate that form of buying. Thus he would buy the $25 worth nine and three-fifth times during twelve months, with a saving of $1.25 each time or a total saving of $12 on an investment of $25, because, as we have seen, $25 is all that he has invested. This is a total annual profit of 48 per cent. on the capital involved. Surely the man who neglects this is little less than financially asleep. But great as this is, it is not all. For with $25 of purchasing capital, he may take advantage of quantity rates on certain staple lines of supplies. A certain widely used line of amalgam is quoted at $2.50 per single ounce, 5 ounces for $10. This means that if Dr. A buys 5 ounces at a time on a $25 purchase and discounts the bill he pays $1.90 per ounce net, while Dr. B,who buys an ounce at a time and lets the bill run, pays $2.50.

Dr. A thus saves 60 cents an ounce, or nearly 32 per cent. of the cost of the amalgam. The figures for several other lines of supplies in constant use show nearly equal returns. In other words, any dentist who can command $25 and so conduct his collections as to have it back when the supplies are used up can make eight times as much as the bank or any other safe investment can hope to pay him.

Joined with his high rate of savings is linked perfect security. He has his money or his supplies always in his own hand. Better than any mines, oil, rubber or other speculative investments are the returns that lay within his easy reach simply by paying his supply bills and taking his discounts.

It is hardly necessary to go on to figure out the savings resulting from the deposit of $90 with the dealer and the securing, at the very start, of eleven and one-eleventh per cent.

For our average buyer this nets nearly 33 per cent. without taking advantage of any quantity rates. Many of the large buyers make 50 per cent. per annum on these terms. No wonder they prosper and that they often quote fees that are the wonder and despair of competitors.

How simple is the prescription for such profits. It has only three elements.

First. Get your money for what you do.

Second. Save or borrow enough money to discount your bills.

Third. Do not despise small savings.—*Dental Digest.*

